# Case Report: Pathological complete response achieved with neoadjuvant immunochemotherapy in synchronous multiple gastric adenocarcinoma

**DOI:** 10.3389/fimmu.2025.1611281

**Published:** 2025-07-18

**Authors:** Ya-hui Sun, Yan Ma, Liang Chen, Hai-rong Li, Xian-Wen Liang, Xiong-hui He, Ke-jian Zou

**Affiliations:** ^1^ Department of Gastrointestinal Surgery, Hainan General Hospital (Hainan Affiliated Hospital of Hainan Medical University), Haikou, China; ^2^ Department of Hepatobiliary and Pancreatic Surgery, Hainan General Hospital (Hainan Affiliated Hospital of Hainan Medical University), Haikou, China; ^3^ Department of Pathology, Hainan General Hospital (Hainan Affiliated Hospital of Hainan Medical University), Haikou, China

**Keywords:** SMGC, neoadjuvant immunochemotherapy, pathological complete response, immune checkpoint inhibitors, microsatellite instability

## Abstract

Synchronous multiple gastric cancers (SMGC) represent a rare clinical entity with no established treatment guidelines. We report a 76-year-old female with two synchronous poorly differentiated adenocarcinomas (dMMR/MSI-H phenotype) in the gastric lesser curvature, clinically staged as cT4bN2M0. Following three cycles of neoadjuvant immunochemotherapy, the patient demonstrated remarkable tumor regression (RECIST 1.1 partial response) and subsequently underwent R0 distal gastrectomy. Histopathological examination confirmed a pathological complete response (ypT0N0, TRG 0).To our knowledge, this represents the first documented case of SMGC achieving pCR with neoadjuvant immunochemotherapy. Our findings suggest that PD-1 inhibition combined with chemotherapy may induce profound tumor regression in SMGC, even in cases with high tumor burden, potentially converting unresectable to resectable disease. This case provides compelling evidence for incorporating immunotherapy in SMGC management and warrants further investigation through clinical trials.

## Background

Synchronous multiple gastric cancer (SMGC), defined as ≥2 distinct primary gastric malignancies occurring simultaneously (1), accounting for 6%–14% of all gastric cancer cases (2). The pathogenesis involves complex interactions between field cancerization, tumor microenvironment heterogeneity, and genetic predisposition (3–5). Current treatment paradigms extrapolate from solitary gastric cancer protocols, despite evidence suggesting SMGC exhibits more aggressive biology and poorer chemotherapy responses (6, 7).

The advent of immune checkpoint inhibitors (ICIs) has revolutionized management of microsatellite instability-high (MSI-H) gastrointestinal malignancies. While recent trials demonstrate promising efficacy of neoadjuvant immunochemotherapy in gastric cancer (8), SMGC-specific data remains absent due to routine exclusion from clinical studies. This knowledge gap is particularly significant given potential inter-lesional heterogeneity in treatment response.

We present the first documented case of SMGC achieving pathological complete response (pCR) following neoadjuvant PD-1 inhibition combined with chemotherapy, providing critical insights into the management of this challenging clinical scenario.

## Case presentation

A 76-year-old female with 12 months of intermittent epigastric pain with well-controlled type 2 diabetes presented and 10 kg unintentional weight loss. No family history of malignancy was reported.

## Diagnostic evaluation

### Endoscopy

**Figure 1:**
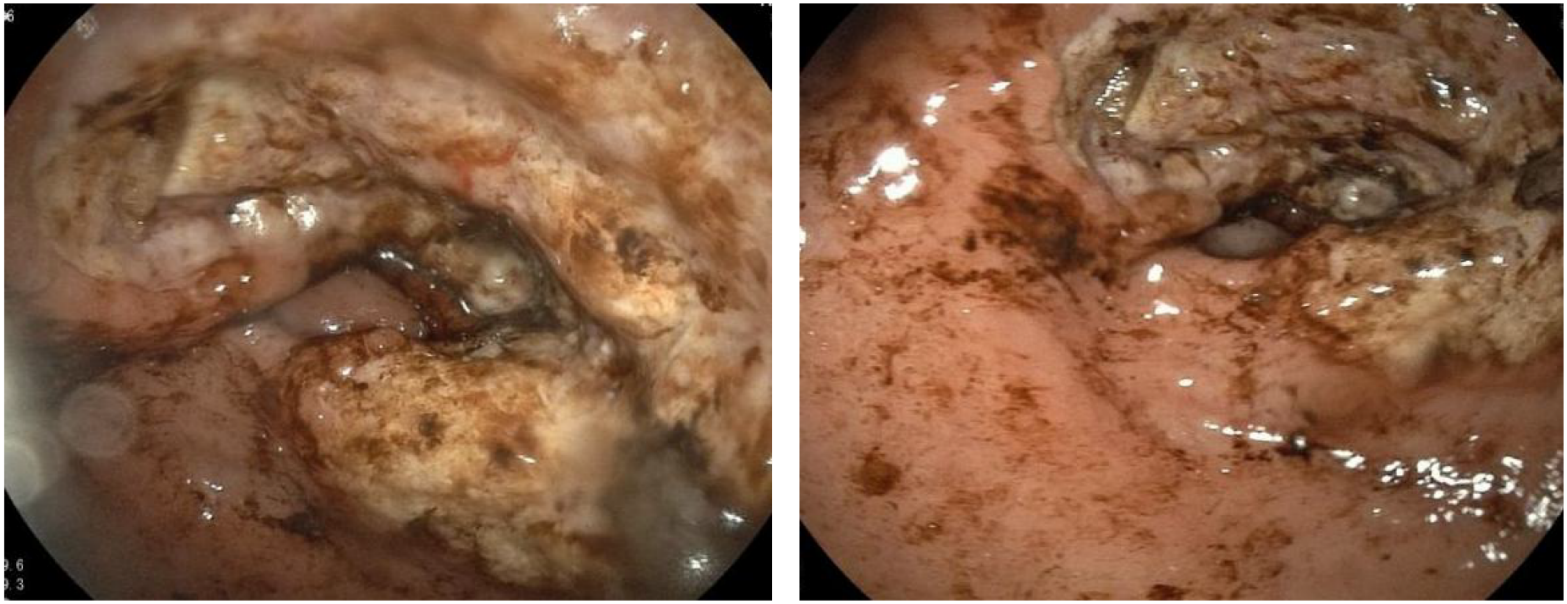
Extensive mucosal ulceration from the lesser curvature of the fundus to the gastric antrum was observed by gastroscopy.

Extensive mucosal ulceration was observed in the lesser curvature to the antrum, with two irregularly elevated ulcerative lesions (**Figure 1**).

### Histopathology

**Figure 2:**
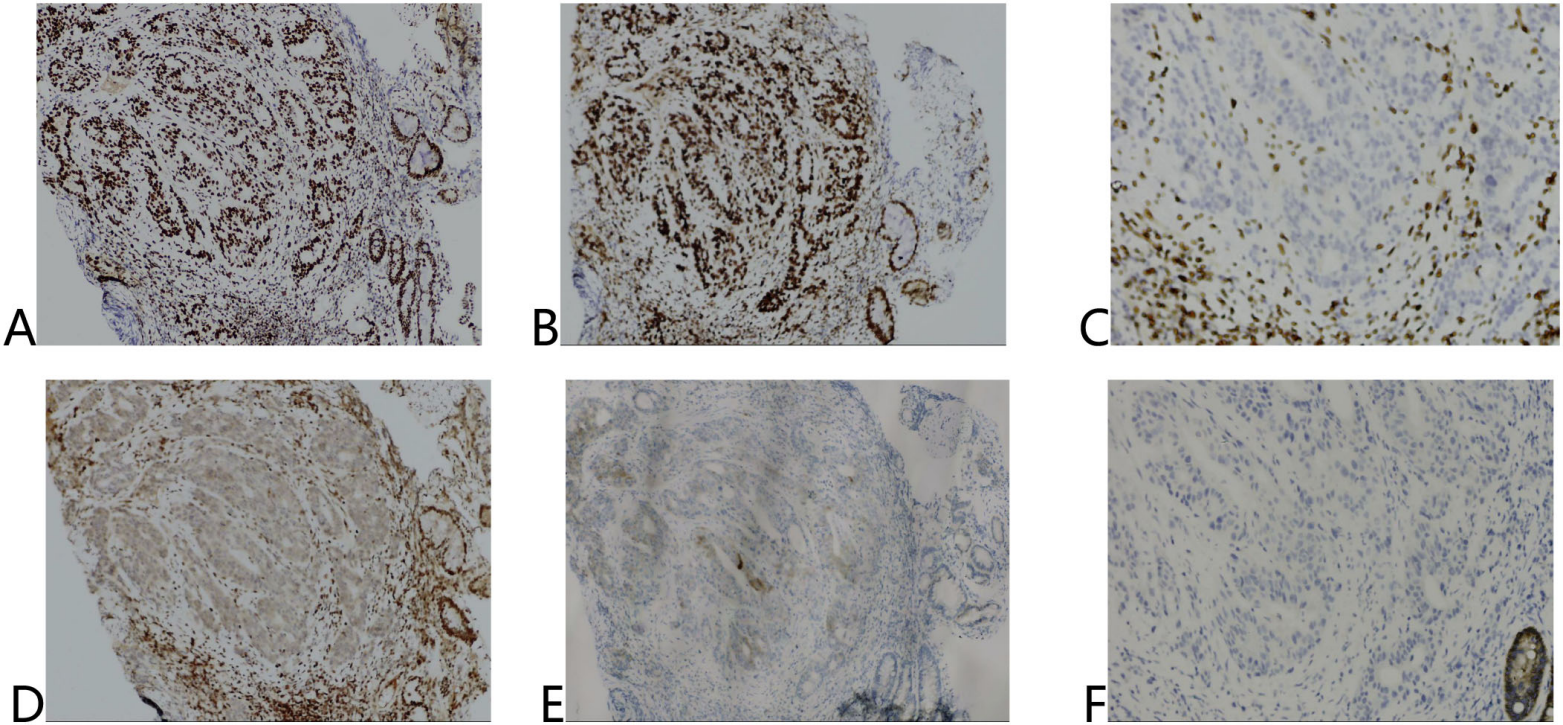
Immunohistochemical results of tumor tissue before treatment. **(A)** MSH2 (+), **(B)** MSH6 (+), **(C)** MLH1 (-), **(D)** PMS2 (-), **(E)** HER2 (1+), **(F)** Claudin18.2 (-).

Both lesions demonstrated poorly differentiated adenocarcinoma (Lauren’s diffuse type) with identical immunohistochemical profiles: MSH2(+), MSH6(+), MLH1(-), PMS2(-), HER2 (1+), Claudin18.2(-) (**Figure 2**).

### Radiological staging (CT)

**Figure 3:**
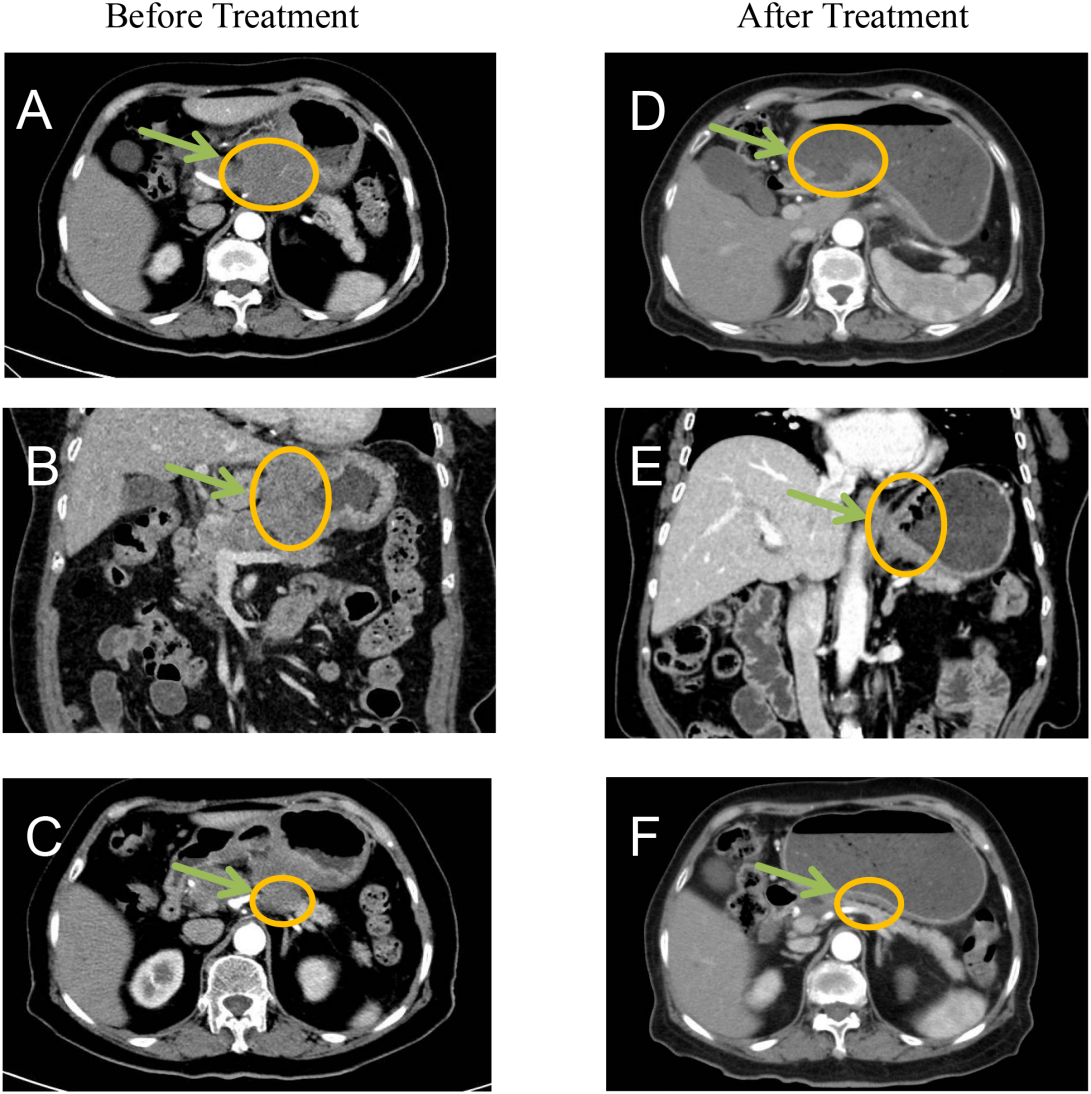
Before treatment CT imaging demonstrated: **(A, B)** Marked thickening and nodularity along the gastric curvature with contrast enhancement, showing poorly defined margins between the stomach and pancreas. **(C)** Significant enlargement of lesser curvature lymph nodes. After treatment imaging revealed: **(D-F)** Substantial reduction in both the primary tumor mass and associated lymphadenopathy, indicating favorable treatment response.

Gastric wall thickening in the lesser curvature with pancreatic invasion, multiple enlarged lymph nodes (maximum: 4.1 cm × 2.7 cm) and no distant metastases. Final clinical stage: cT4bN2M0 (AJCC 8th ed. stage IVA) (**Figures 3A-C**).

### Multidisciplinary decision-making

A multidisciplinary team (MDT) determined that R0 resection was unlikely due to pancreatic involvement and confluent lymph node metastases. The patient received neoadjuvant therapy with SOX (Oxaliplatin + tegafur/gimeracil/octeracil (S-1)) combined with tislelizumab (200 mg on day 1), every 3 weeks, for three cycles, with no significant adverse effects.

### Therapeutic response

Post-treatment CT demonstrated significantly reduction in primary lesions and lymph node (**Figures 3D-F**).

### Surgical intervention

**Figure 4:**
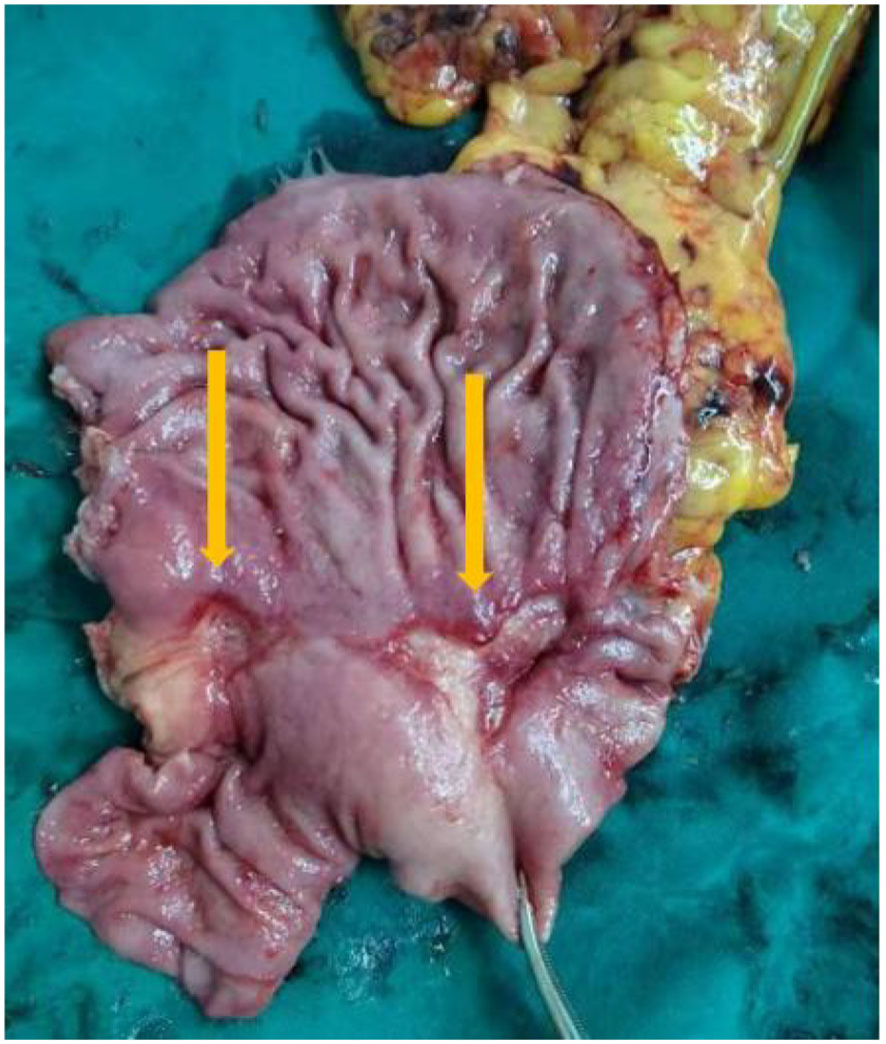
Surgically removed specimens showed two ulcers in the lesser curvature of the stomach and significant receding scars after neoadjuvant therapy.

Laparoscopic distal gastrectomy with D2 lymphadenectomy (R0) was performed. Intraoperative findings revealed Two fibrotic ulcer beds (1.7cm×2.4 cm; 3.8cm×2.1 cm) with significant post-treatment scarring (**Figure 4**).

### Pathological evaluation

**Figure 5:**
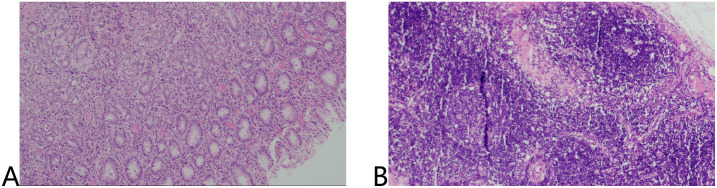
HE staining of surgical specimens including tumor tissue **(A)** and lymph nodes **(B)**.

No residual cancer cells were detected in the ulcers or lymph nodes (ypT0N0). Tumor regression grade (TRG): 0 (Ryan criteria) (**Figure 5**).

## Discussion

Comprehensive genomic profiling has established gastric cancer as a molecularly heterogeneous disease comprising distinct subtypes, each exhibiting unique molecular characteristics and clinical behaviors. Per the Cancer Genome Atlas (TCGA) classification system, gastric cancer can be categorized into four molecular subtypes: microsatellite instability (MSI), chromosomal instability (CIN), Epstein-Barr virus (EBV)-positive, and genomically stable (GS) tumors (9, 10). Of these, the MSI subtype has emerged as a particularly noteworthy entity.

Microsatellites (MS), defined as short, repetitive DNA sequences ubiquitously distributed throughout the human genome, are highly prone to replication errors (11). The DNA mismatch repair (MMR) system serves as the primary mechanism for detecting and correcting such errors. Consequently, genetic or epigenetic alterations in MMR genes may compromise MMR function (dMMR), thereby inducing a high microsatellite instability (MSI-H) phenotype. This molecular signature is associated with genomic instability and an increased tumor mutational burden (12–14).

The advent of immune checkpoint inhibitors (ICIs) targeting programmed death-1 (PD-1) and programmed cell death ligand 1 (PD-L1) has revolutionized cancer treatment paradigms (9). Accumulating evidence has demonstrated a strong association between MSI status and ICI efficacy, and more and more studies have begun to pay attention to the effect of ICIs in neoadjuvant therapy for gastric cancer (15, 16).

Notably, the recently published NEOSUMMIT-01 trial reported a pathological complete response (pCR) rate of 22.2% in locally advanced gastric cancer patients receiving neoadjuvant immunochemotherapy (the PD-1 inhibitor tislelizumab plus SOX regimen), representing a significant improvement over chemotherapy alone (7.4%) (8). However, this study specifically excluded patients with SMGC, leaving the efficacy of immunotherapy in this population unexplored.

To our knowledge, this represents the first documented case of SMGC achieving pCR following neoadjuvant immunochemotherapy. Notably, despite presenting with extensive lymph node metastasis at diagnosis, postoperative pathological examination revealed complete tumor regression, suggesting that immunotherapy may eradicate micrometastases through systemic immune activation. Intraoperative findings demonstrated significant fibrosis along the lesser curvature, potentially attributable to immunotherapy-induced fibroblast activation and collagen deposition. While these changes may obscure surgical planes and increase procedural complexity, they are considered favorable prognostic indicators. Furthermore, current evidence indicates that cancer stage, rather than lesion multiplicity, serves as the primary determinant of SMGC prognosis (17).

In the present case, the achieved pathological pCR following immunotherapy may be associated with multiple factors including systemic immune activation, elevated tumor mutational burden (TMB), MSI-H status, and dynamic tumor microenvironment remodeling. Moreover, the establishment of immunological memory might facilitate eradication of minimal residual disease and potentially mitigate recurrence risk.

Extensive research has been conducted on laparoscopic gastrectomy following neoadjuvant chemotherapy for gastric cancer. Although neoadjuvant chemotherapy induces significant tissue edema and fibrosis, increasing surgical complexity, advancements in surgical instrumentation (e.g., ultrasonic dissectors) and refined operative techniques have substantially minimized iatrogenic damage to normal tissues (18). The safety and feasibility of this approach have been robustly validated in multiple clinical studies.

## Data Availability

The original contributions presented in the study are included in the article/**Supplementary Material**. Further inquiries can be directed to the corresponding authors.
