# Supplementary figures and images for: Case Report: Pathological complete response achieved with neoadjuvant immunochemotherapy in synchronous multiple gastric adenocarcinoma
Source: Front Immunol. 2025 Jul 18;16:1611281. doi: 10.3389/fimmu.2025.1611281 (PMC12313488; doi:10.3389/fimmu.2025.1611281)

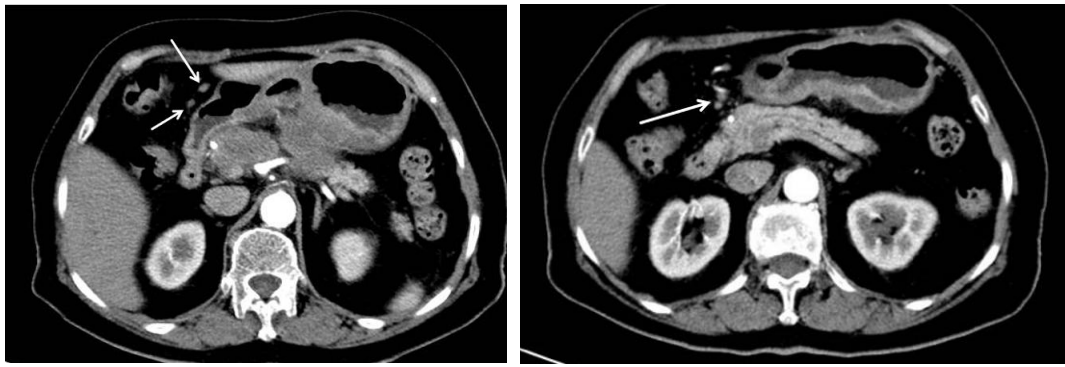

CT images of enlarged lymph nodes

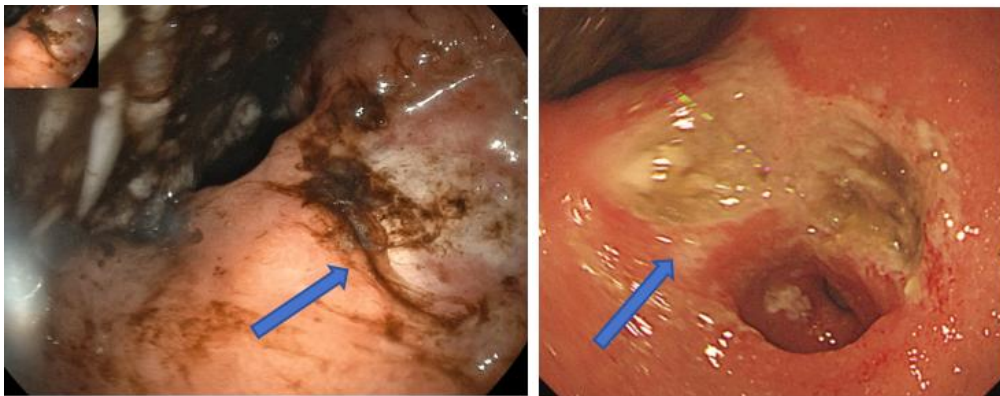

Gastroscopy images show multiple mucosal ulcers

Supplement: Supplementary file 1 [file DataSheet1.pdf]
