# Supplementary material for: Case Report: Pathological complete response achieved with neoadjuvant immunochemotherapy in synchronous multiple gastric adenocarcinoma
Source: Front Immunol. 2025 Jul 18;16:1611281. doi: 10.3389/fimmu.2025.1611281 (PMC12313488; doi:10.3389/fimmu.2025.1611281)
Supplement: Supplementary file 2 [file DataSheet2.pdf]

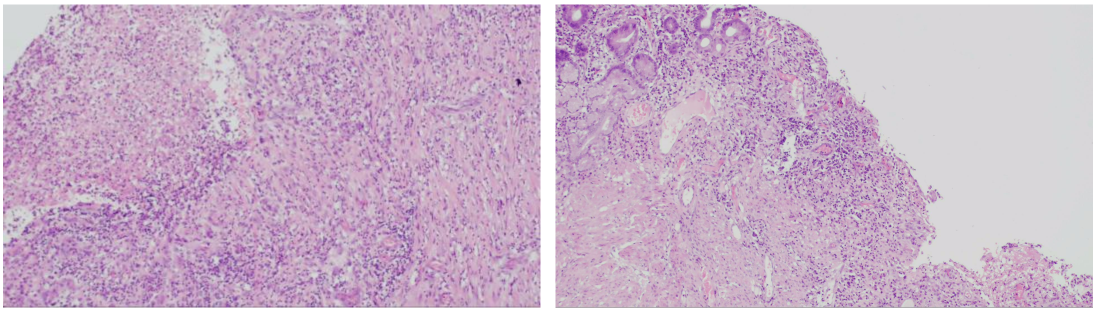

HE staining of two ulcers in the postoperative specimens

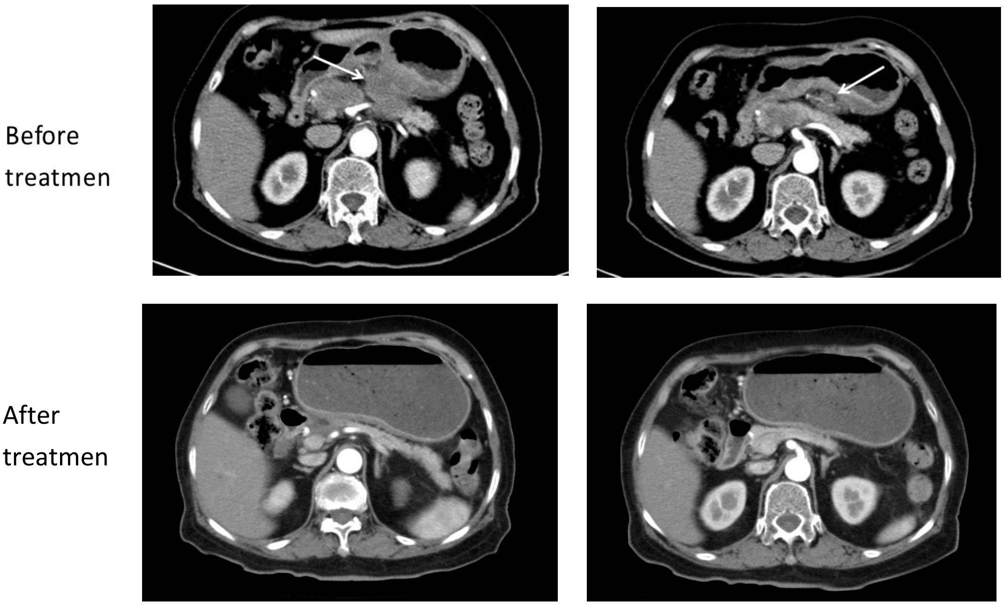

Before the treatment, the lymph nodes on the lesser curvature of the stomach were significantly enlarged and had uneven density. After the treatment, no obviously enlarged lymph nodes were observed at the same site

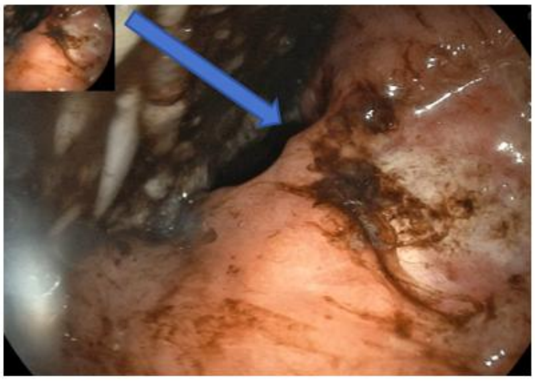

The arrow points to the oral end of the tumor
